# Editors' Wishes for an Illuminated Season and an Open New Year

**DOI:** 10.1371/journal.pmed.1001579

**Published:** 2013-12-31

**Authors:** The PLOS Medicine Editors

## Abstract

In their editorial for December 2013, the *PLOS Medicine* Editors reflect on recent advances in Open Access to medical research and identify challenges to be addressed in 2014.

*Please see later in the article for the Editors' Summary*

For our readers who find their way to this *PLOS Medicine* editorial on its publication day, it will be New Year's Eve, that traditional boundary between past and future. Perhaps this holiday is itself a precursor of medical research, being an example of how careful observation of nature shapes human activity; it comes on a day when ancient empiricists (in the Northern hemisphere, anyway) might have felt relief at good evidence that winter days were indeed going to get lighter.

It's been a busy season for extending the light of scientific understanding through access to its reports. Broadly conceived, scientific research can explore the universe from its greatest expanses to it minutest components, but medical research, if it is to be worthy of the name, has to find its way back to the human body in its dignities and privacies, the mind's motivation towards meaningful activity, and the intricate social institutions that shape our responses to illness. Looking forward, we see great opportunities for Open Access publications to advance human health, provided the medical research and publishing communities can rise to the challenges that come with them.

## “Social” Interactions around Medical Research

Whatever journal editors may prefer, it is simply a fact that post-publication peer review can take place in any number of locations, most of which are remote from the journal-based article. The biggest holiday event on this theme has to have been the launch of PubMed Commons, which permits authors with certain qualifications to comment on PubMed abstracts. As the initial “closed beta pilot commenting system” transitions to more general availability, stakeholders will continually encounter the challenge of how to ensure that commentary raises the standard of discourse (without, for example, presenting marketing messages outside the peer-review process). It is also unclear how comments will address full publications rather than abstracts alone, particularly when the full article is not openly available. Journals will be able to import comments into their own article display, but will need to address technical challenges in order to do so. Since commenting itself is a social process, it seems natural that reactions to the launch of PubMed Commons were “storified” [1], blogged and widely retweeted. In the dawning age of social media, where is one best advised to look for scientific advances? Probably never again in just one or two places.

## Access to Results of Clinical Research

The importance of looking beyond a single journal article is also suggested by a study published earlier this month in *PLOS Medicine* by Dechartres and colleagues, who searched trial registrations in the ClinicalTrials.gov database and found that, among trial registrations with results posted in the database, only half also had trial results published in a peer-reviewed journal [2]. For those trials with results in both locations, they found that ClinicalTrials.gov reported efficacy results, the flow of participants, and adverse events significantly more often than did published articles. Thus, the availability of trial results in ClinicalTrials.gov supplements, and in some cases supplants, the journal article as the source for clinical trials results. While Clinicaltrials.gov has become an important source for trial information, peer review remains an important step in presenting reliable interpretations of trial results. Open availability of trial results in public databases provides a constructive challenge for editors, authors, and peer reviewers to improve the comprehensiveness and validity of formally published results.

## Exposing and Addressing Bias

Among our research articles this month is a “systematic review of systematic reviews” by Bes-Rastrollo and colleagues [3], which suggests that systematic reviews with stated sponsorship by, or conflicts of interests related to, food companies are more than five times more likely to report a non-positive association between sugar-sweetened beverage consumption and weight gain or obesity than those without any statement about sponsorship or conflicts of interest. This study adds a link to the chain of evidence that conflict of interest statements alone cannot mitigate the potential for bias and are at best an imperfect solution to protect the integrity of research. Shining a light on entrenched conflicts of interest that threaten the integrity of medical research, and identifying effective solutions, remain priorities for *PLOS Medicine* in 2014.

Publication bias also remains a problem, as many clinical studies are never published, for reasons ranging from intent to suppress “undesirable” results to loss of interest in writing and submitting the manuscript. Not publishing ethically conducted research is an ethical breach in itself: patients participate with the understanding that study results will be shared. Publishing all ethically conducted trials regardless of results is the goal of the AllTrials initiative, and *PLOS Medicine* has taken steps to help bring that goal to fruition, through the RIAT initiative (Restoring Invisible and Abandoned trials) [4],[5], and, under specific circumstances, removing the editorial requirement for prospective trial registration, provided that authors explain why the trial was not prospectively registered, register their trial and any related trials, and post their trial results on ClinicalTrials.gov or other registry at least by the time of article publication [6]. As with any other studies published in *PLOS Medicine*, they must also meet the requirements of the PLOS data sharing policy [7]. While the latest version of the Declaration of Helsinki [8], released after *PLOS Medicine*'s change in editorial policy, suggests that journal editors should not accept for publication trials (or indeed, any prospective studies) that have not been prospectively registered, adhering to the letter of such a requirement would ensure that some trials, even those conducted ethically and appropriately but with some misunderstanding, lack of knowledge, or administrative glitch that resulted in retrospective registration, would never see the light of day in a peer-reviewed journal. We believe that *PLOS Medicine*'s policy supports the goal of the Declaration of Helsinki—ethical principles for medical research involving human subjects, including the Declaration's statement that “Researchers have a duty to make publicly available the results of their research on human subjects and are accountable for the completeness and accuracy of their reports”—even more effectively by, under specific conditions, considering retrospectively registered clinical trials for possible publication, and at the same time encouraging registration of all types of clinical research and public posting of study results.

## Data Sharing

In a *PLOS Medicine* Policy Forum this month, Karunakara and colleagues report on their experience sharing data from projects conducted by Médecins Sans Frontières (MSF) [9]. Despite the challenges of political or ethnic violence, government restrictions, or potentially dangerous consequences for individuals with certain disease diagnoses, MSF has managed to find a way to share data while ensuring that patients are not harmed or compromised. If MSF can rise to this challenge and safely share data under such difficult circumstances, research enterprises in Europe and North America should be able to find the wherewithal to share data safely and maximize the potential benefit of research for everyone.

## Crowdsourcing the Magnitude of the Paywall Problem

Even as PLOS celebrates 10 years as an Open Access publisher, and mandates by government and funding agencies establish Open Access as the norm for publishing research [10], subscription barriers continue to impede scientific progress and access to critical information in the medical literature. Since most encounters with a subscription barrier occur as silent moments of frustration for individual readers, the magnitude of these barriers has been difficult to quantify. The student-led Open Access Button project aims to provide real-time, worldwide, interactive insight into this problem. The project is based on a simple browser plug-in, which will record when a paywall is encountered, the reader's location and profession, and the reason for wanting to access the article. The plugin will also search for any freely accessible version of the article. A beta version of the tool was released in November and will provide fresh data on the impact of further expanding Open Access.

The *PLOS Medicine* Editors are glad of the progress that has taken place in 2013 and look forward to the support of our authors, academic editors, peer reviewers, and readers in addressing the challenges of the New Year. Good health!
